# Smoking Dependent Alterations in Bone Formation and Inflammation Represent Major Risk Factors for Complications Following Total Joint Arthroplasty

**DOI:** 10.3390/jcm8030406

**Published:** 2019-03-24

**Authors:** Sabrina Ehnert, Romina H. Aspera-Werz, Christoph Ihle, Markus Trost, Barbara Zirn, Ingo Flesch, Steffen Schröter, Borna Relja, Andreas K. Nussler

**Affiliations:** 1 Department of Trauma and Reconstructive Surgery, Siegfried Weller Research Institute, Eberhard Karls University Tuebingen, BG Trauma Center Tuebingen, 72076 Tuebingen, Germany; sabrina.ehnert@med.uni-tuebingen.de (S.E.); rominaaspera@hotmail.com (R.H.A.-W.); trost.m@gmx.net (M.T.); zirn.b@gmx.de (B.Z.); iflesch@bgu-tuebingen.de (I.F.); sschroeter@bgu-tuebingen.de (S.S.); andreas.nuessler@gmail.com (A.K.N.); 2 Department of Trauma, Hand and Reconstructive Surgery, University Hospital Frankfurt, Goethe University, 60590 Frankfurt, Germany; info@bornarelja.com

**Keywords:** total joint arthroplasty (TJA), cigarettes, smoking, pack-years (PY), complications, infection, delayed wound healing, revision surgery, bone metabolism

## Abstract

Numerous studies have described a correlation between smoking and reduced bone mass. This not only increases fracture risk but also impedes reconstruction/fixation of bone. An increased frequency of complications following surgery is common. Here, we investigate the effect of smoking on the clinical outcome following total joint arthroplasty (TJA). 817 patients receiving primary or revision (including clinical transfers) TJA at our level-one trauma center have been randomly interviewed twice (pre- and six months post-surgery). We found that 159 patients developed complications (infections, disturbed healing, revisions, thrombosis, and/or death). Considering nutritional status, alcohol and cigarette consumption as possible risk factors, OR was highest for smoking. Notably, mean age was significantly lower in smokers (59.2 ± 1.0a) than non-smokers (64.6 ± 0.8; *p* < 0.001). However, the number of comorbidities was comparable between both groups. Compared to non-smokers (17.8 ± 1.9%), the complication rate increases with increasing cigarette consumption (1–20 pack-years (PY): 19.2 ± 2.4% and >20 PY: 30.4 ± 3.6%; *p* = 0.002). Consequently, mean hospital stay was longer in heavy smokers (18.4 ± 1.0 day) than non-smokers (15.3 ± 0.5 day; *p* = 0.009) or moderate smokers (15.9 ± 0.6 day). In line with delayed healing, bone formation markers (BAP and CICP) were significantly lower in smokers than non-smokers 2 days following TJA. Although, smoking increased serum levels of MCP-1, OPG, sRANKL, and Osteopontin as well as bone resorption markers (TRAP5b and CTX-I) were unaffected. In line with an increased infection rate, smoking reduced 25OH vitamin D3 (immune-modulatory), IL-1β, IL-6, TNF-α, and IFN-γ serum levels. Our data clearly show that smoking not only affects bone formation after TJA but also suppresses the inflammatory response in these patients. Thus, it is feasible that therapies favoring bone formation and immune responses help improve the clinical outcome in smokers following TJA.

## 1. Introduction

Smoking indisputably affects human health, e.g. increases the risk of cancers, cardiovascular and respiratory diseases, reproductive problems, and other medical maladies. However, one of the little known effects of smoking is that on injuries. A correlation between cigarette consumption and reduced bone mass was described for the first time in 1976 [1]. This correlation has been confirmed in several epidemiologic studies since then [2,3]. Consequently, smokers seem to be at higher risk for fragility fractures [4,5]. For example, in patients with distal radius fractures, smokers show more frequently post-surgical tenderness, wrist stiffness, non-union, and revision surgeries as compared to non-smokers [6]. Similarly, Pearson et al. reported not only a significant delay in fracture healing, but also a two-fold increase in the risk for developing a non-union after fracture, spinal fusion, osteotomy or arthrodesis in smokers [7]. In orthopedic surgeries, smokers frequently show delayed fracture healing, higher frequency of complications, and prolonged hospital stays as compared to non-smokers [8,9]. This might be partly due to alterations in bone density and structure, which in turn impede reconstruction and fixation of the bone. However, the underlying mechanisms affecting bone quality are not yet fully understood.

Bone metabolism in smokers can be affected either directly via toxic effects on the bone cells or indirectly via changes in hormone status, vasculature, immune responses, or oxygenation [8,10]. This in turn may compromise wound and fracture healing. Most research has been done on bone loss in the jaw of smokers with periodontitis. In these patients, serum levels of osteoprotegerin (OPG) are reduced while serum levels of sRANKL (soluble receptor activator of nuclear factor kappa-B ligand) are unchanged, proposing an increased bone turnover [11,12,13,14,15,16,17]. However, it is questionable if these observations can be translated to skeletal bone. In the oral cavity, effects on bone are not only mediated by factors distributed via the blood stream (as with most sites of skeleton) but may, to a large part, be mediated by direct toxic effects following contact with the cigarette smoke. Osteogenic differentiation of bone marrow-derived mesenchymal stem cells, for example, is inhibited when exposed to cigarette smoke extract [18,19]. Furthermore, primary human osteoblast viability is strongly affected when exposed to cigarette smoke extract [20]. Oxidative stress, which is well accepted to be increased in smokers, seems to be a key regulator for this.

Oxidative stress may cause increased synthesis of monocyte chemoattractant protein 1 (MCP-1), also known as C-C motif chemokine 2 (CCL2), an important chemotactic factor for monocytes and macrophages [21]. It has been shown that MCP-1 is instrumental in favoring the formation of osteoclasts [22,23,24,25]. There are reports, showing that MCP-1 may favor osteoclast fusion and osteoclastogenesis both in an autocrine and paracrine manner [23]. Expression of MCP-1 by osteoclasts is proposed to be regulated by sRANKL [24]. Similarly, 25 hydroxy vitamin D_3_ (25(OH)D_3_) and its metabolite 1,25 dihydroxy vitamin D_3_ (1,25(OH)_2_D_3_) were identified as key regulators of MCP-1, OPG, and sRANKL in this process [26,27]. However, regulation of the individual genes is different: MCP-1 expression is reported to be reduced by 25(OH)D_3_ [28], and sRANKL expression is reported to be increased by 25(OH)D_3_ [27]. Paradoxically, supplementation with vitamin D_3_ improves bone mineral density in patients via inhibition of osteoclasts [26,29]. Both insufficient uptake or synthesis of vitamin D and insufficient processing are supposed to decrease 25(OH)D_3_ and 1,25(OH)_2_D_3_ serum levels in smokers [30], which in turn might favor bone resorption [31]. However, 25(OH)D_3_ is also known as a potent regulator of immune responses [32].

In the oral cavity of smokers (with and without periodontitis), inflammatory markers, e.g. IL-1β, IL-6, or TNF-α, are frequently increased [33,34]. This is in clear contrast to reports describing a general immune suppression in smokers [35,36,37], affecting their ability to fight infections. Indeed, epidemiologic studies suggest that smokers are at higher risk for developing infections following injuries [30]. Besides the ability to fight infections, an ongoing inflammation response in the early fracture hematoma is required to induce fracture healing [38].

Although it is well described that smoking increases the risk for fractures and complications during the following healing process, little is known about the possible underlying mechanisms and thus specific treatment options. Despite great progress in implant and surgical technologies, handling of smokers during and after orthopedic and trauma surgeries remains a huge challenge. Therefore, we wanted to first confirm that smoking is a major risk factor for complications (infections, disturbed wound healing, required revision surgery, thrombosis, and/or death) up to six months following surgery in our study cohort comprised of 817 both in-house and transfer patients that received a primary or revision total joint arthroplasty (TJA). Then, we conducted a closer investigation of the study cohort regarding age, gender, BMI, complications, and duration of hospital stay, with respect to the amount of cigarettes consumed. In order to identify possible underlying mechanisms for delayed bone healing and infections, we measured blood serum markers for bone formation and resorption, oxidative stress, and inflammation two days post-surgery in non-, moderate, and heavy smokers. Based on the available literature, we hypothesize a shift in bone metabolism in smokers, characterized by a decrease in bone formation markers, as well as an increase in oxidative stress and osteoclast markers. Furthermore, a decreased inflammatory response following surgery is expected based on the proposed higher complication rate in smokers.

## 2. Experimental Section

### 2.1. Ethics Statement

The study, including patient material, was performed in accordance with the Declaration of Helsinki (1964) in its latest amendment. Patient survey and collection of the clinically relevant data was performed in accordance with the ethical vote 193/2014BO2. Additional blood sampling was performed during a routine blood sampling (ethical vote 538/2016BO2). All study participants have signed a written informed consent.

### 2.2. Patient Recruitment and Survey

Study participants were recruited between June 2014 and January 2018. A consecutive series of patients at our arthroplasty center of maximum care as well as patients that were transferred to our septic surgery department from other hospitals because of complications following TJA of the hip or knee have been included. Patients who were hospitalized for two or more nights and agreed to participate in the presented study were included. Patients suffering from dementia, patients with insufficient knowledge of the study language, and patients who could not answer the questions because of severe health conditions were excluded. All patients were screened at hospital admission during a bedside interview and in telephone interviews in the following six months. To avoid observer-dependent bias, all observers were trained for two weeks. General patient data and nutritional status as well as alcohol and cigarette consumption were detected. After discharge (in general, 12–14 days after surgery and initial mobilization with the in-house physiotherapist), complications were captured out of the hospital information system. As described in a prior publication of our working group, complications were defined as death, infections, wound healing disorders, further operations, and thrombosis [39,40]. All adverse events were weighted equally and assessed during hospitalization as well as six months post-surgery.

### 2.3. Blood Sampling

A 10 ml blood sample (5 ml serum and 5 ml EDTA) was obtained during a routine blood sampling. Blood samples were centrifuged at 1000 *g* for 10 min at room temperature within a time frame of 30 min after sampling. Resulting serum and plasma samples were stored in aliquots at −80 °C until further use.

### 2.4. Cytokine Array

To determine relative serum levels of cytokines, a RayBio^®^ Human Cytokine Array C5 (BioCat, Heidelberg, Germany) was performed according to the manufacturer’s instructions. For the signal development, the dot blot membranes were incubated for 1 min with ECL substrate solution. Chemiluminescent signals were detected with a CCD camera (INTAS, Göttingen, Germany). Signal intensities were measured using the ImageJ software (NIH, Bethesda, MA, USA). On each array membrane, the 6 spots (positive controls) were used for normalization.

### 2.5. Enzyme Linked Immunosorbent Assay (ELISA)

Target proteins in serum samples were quantified with the help of ELISA kits, performed as indicated by the manufacturer. An overview is given in Table 1.

### 2.6. Statistics

Distributions within groups are represented as Venn diagrams, pie charts, or contingency tables. Results are represented either as box blots (Box and Whiskers–Tukey to visualize outliers) or as scatter diagrams (mean ± 95% confidence interval). The number of donors (*N*) and technical replicates (*n*) is given in the figure legends. Comparison of multiple groups was done using the Kruskal–Wallis H-test followed by Dunn’s multiple comparison test. The Mann–Whitney U-test (2-sided) was used to compare two single groups with each other. Data are summarized as mean ± SEM; 95% confidence interval. Statistical analysis was performed using the GraphPad Prism Software (Version 5, El Camino Real, California, CA, USA). *p* < 0.05 at an α = 0.05 was taken as minimum level of significance.

### 2.7. Data Availability

The datasets generated and analyzed during this study are available from the corresponding author upon reasonable request.

## 3. Results

### 3.1. Patient Recruitment and Description of The Study Cohort

In total, 817 patients (359 women and 458 men) receiving TJA (primary or revision), including patients that were transferred to our hospital because of post-surgical complications, were randomly interviewed for this study. However, 29 patients were lost to follow up because of missing data sets.

**Figure 1:**
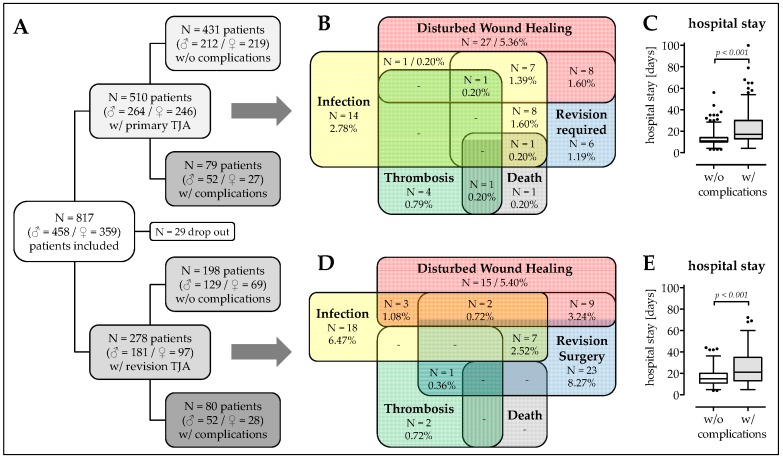
Overview on the study population. (**A**) Flow chart on the patient recruitment: Between June 2016 and January 2018, a total of 817 patients with total joint arthroplasties (TJA) were interviewed for our study. However, 29 patients had to be excluded from the study because of missing data sets. Of the remaining 788 patients, 510 (62.4%) received primary TJA and 284 (37.6%) had a revision TJA. Of the patients with primary TJA, 431 (84.5%) had no complications and 79 (15.5%) had complications up to six months following surgery. Of the patients with revision TJA, 189 (71.2%) had no complications and 80 (28.8%) had complications up to six months following surgery. Venn diagrams on the complications, with the number in patients affected (N) and the relative occurrence (in %) with regard to (**B**) the patients with primary TJA and (**D**) the patients with revision TJA. Duration of hospital stay (in days) of the study participants with (**C**) primary TJA and (**E**) revision TJA.

Overall, 510 patients received primary TJA and 278 patients received revision TJA. In both groups together, 159 patients developed complications (infections, disturbed wound healing, required revision surgery, thrombosis, and/or death) within six months following surgery (Figure 1A). The complication rate was significantly lower in patients with primary TJA (15.5%; Figure 1B) than in patients with revision TJA (28.8%; OR = 2.204; *p* = 0.009; Figure 1D). As expected, patients with complications stayed significantly longer in hospital (Primary: 24.1 ± 18.9 days; 19.9–28.4 days. Revision: 25.7 ± 16.3 days; 22.1–29.4 days) than patients that did not develop adverse events (Primary: 12.6 ± 5.6 days; 12.1–13.2 days; *p* < 0.001. Revision: 16.6 ± 7.6 days; 15.5 – 17.6 days; *p* < 0.001; Figure 1C,E).

Differentiating between primary and revision TJA, the mean age was comparable between the group that developed complications (59.9 ± 14.7a) and the group that did not develop complications (Primary: Δ_mean_ 2.3a; *p* = 0.106. Revision: Δ_mean_ 0.4a; *p* = 0.437). When considering primary TJA, there were more male patients in the group with complications (65.8%) than in the group without complications (49.2%; *p* = 0.022). Interestingly, this imbalance in gender distribution was not present in patients receiving revision TJA. For both primary and revision TJA, BMI was comparable between patients developing or not complications (Primary: Δ_mean_ 0.9 kg/m^2^; *p* = 0.263. Revision: Δ_mean_ 0.8 kg/m^2^; *p* = 0.809). Similarly, the number of comorbidities (primary: Δ_mean_ 0.39; *p* = 0.821/revision: Δ_mean_ 0.29; *p* = 0.574) and medication (Primary: Δ_mean_ 0.69; *p* = 0.844. Revision: Δ_mean_ 0.01; *p* = 0.795) were comparable between both groups. Interestingly, while in patients receiving primary TJA, the frequency of malaise (nausea and/or vomitus) prior surgery only trends to be higher in the group that developed complications (17.7%) when compared with the group that did not develop adverse events (9.3%; OR = 2.220; *p* = 0.097), this effect was highly significant in patients receiving revision TJA (8.6% *vs.* 31.3%; OR = 4.543; *p* < 0.001). For overview, see Table 2.

### 3.2. Higher Frequency of Malnourished Patients and Smokers in The Complication Group

**Figure 2:**
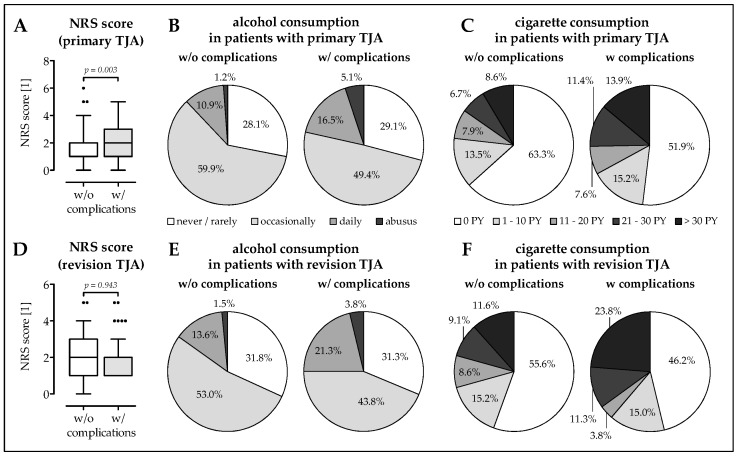
Nutritional status and alcohol and cigarette consumption in patients receiving (**A**–**C**) primary TJA or (**D**–**F**) revision TJA. (**A**,**D**) Nutritional status was obtained with the help of the nutrition risk screening 2002, which defines a nutritional risk for obtained scores ≥3. The data are presented as box blot Tukey to mark ouliers (*). (**B**,**E**) Pie diagrams showing the alcohol consumption in the study population. Alcohol consumption was defined as never/rarely, occasionally, daily (one glass of wine or beer), and abuse (daily more than one glass of wine, beer and/or hard liquor). (**C**,**F**) Pie diagrams showing the smoking behavior of the study population. Cigarette consumption was measured in pack-years (PY), with the number of PY being ((number of cigarettes smoked per day/20) × (number of years smoked)).

The risk for malnutrition was determined with the help of the nutritional risk screening 2002 questionnaire (NRS) [40]. Patients who developed complications following primary TJA scored significantly higher (2.03 ± 1.04; 1.78–2.26; *p* = 0.003) than patients who did not develop complications (1.65 ± 0.93; 1.56–1.74; Figure 2A). There was a higher frequency of patients (primary TJA) at risk for malnutrition (NRS ≥ 3) in the group developing complications, resulting in an OR of 2.315 (*p* = 0.026). Interestingly, the observed difference in NRS score (and frequency of malnutrition) was not existent in patients with revision TJA (1.90 ± 0.99; 1.76–2.04 vs. 1.91 ± 0.98; 1.69–2.13), which already had a higher NRS in the group without complications (Figure 2D). In both primary and revision TJA, the rate of daily alcohol consumption and alcohol abuse is higher in the group with complications than in the group without complications, without a marked difference between patients receiving primary or revision TJA (Figure 2B,E). More pronounced was the difference in smoking behavior. There was a clear difference between the patients receiving primary and revision TJA observed. Overall, patients who received a primary TJA smoked less than patients who received a revision TJA. In this group, patients who did not develope a complication had comparable smoking behavior to patients receiving a primary TJA but developed a complication. Overall, the proportion of non-smokers was lower in the group without complications. The proportion of moderate smokers (1–20 pack-years [PY]) was comparable between all four groups investigated. The rate of heavy smokers was almost twice as high in the group with complications as in the group without complications (Figure 2C,F). In line with this, the determined odds ratios increase with increasing PY. For primary TJA: (i) >0 PY: OR = 1.601, (ii) >10 PY: OR = 1.624, and (iii) >20 PY: OR = 1.875; *p* = 0.034. For revision TJA: (i) >0 PY: OR = 1.453, (ii) >10 PY: OR = 1.527, and (iii) >20 PY: OR = 2.062; *p* = 0.015. Interestingly, there was no significant difference in the rate of active smokers to former smokers between the four groups investigated.

### 3.3. Smokers Have More Complications at a Younger Age

**Figure 3:**
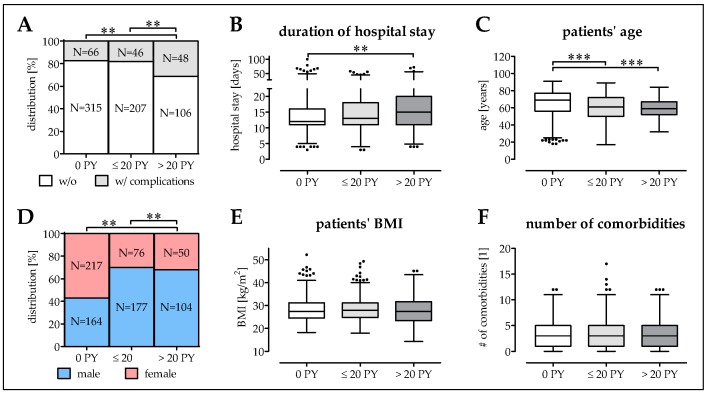
Description of the study population based on the smoking behavior. The analysis differentiated between non-smokers (0 PY; *N* = 381), moderate smokers (1–20 PY; *N* = 253) and heavy smokers (>20 PY; *N* = 154). (**A**) Complication rate is given in % and total numbers (*N*). (**B**) Time of hospitalization was documented in days. (**C**) Patients’ age is given in years. (**D**) Gender distribution within the groups is given in % and total numbers (*N*). (**E**) Patients’ BMI is given in kg/m^2^. (**F**) Number of comorbidities. * *p* < 0.05, ** *p* < 0.01, and *** *p* < 0.001 as indicated.

We investigated the effect of smoking in the clinical outcome in our patient cohort. Compared to non-smokers (17.3 ± 1.9%; 13.5–21.1%), the complication rate was not significantly increased in moderate smokers (1–20 PY: 18.2 ± 2.4%; 13.4–23.0%) but almost doubled (31.2 ± 3.7%; 23.8–38.6%; *p* ≤ 0.004) in heavy smokers (≥20 PY; Figure 3A). Consequently, the mean hospital stay for heavy smokers was significantly longer (18.4 ± 1.0 days; 16.4–20.4 days) compared to non-smokers (15.3 ± 0.5 days; 14.3–16.4 days; *p* = 0.009) or moderate smokers (15.9 ± 0.6 days; 14.8–17.1 days; Figure 3B). It is noteworthy that the mean age of moderate smokers (59.1 ± 1.0a; 57.2–61.1a; *p* < 0.001) and heavy smokers (59.3 ± 0.9a; 57.2–61.2a; *p* < 0.001) was significantly lower than the mean age of non-smokers (64.6 ± 0.8a; 63.0–66.2a; Figure 3C). Overall, smoking was more prominent in male patients than female patients (69.0% male smokers vs. 43.0% male non-smokers; *p* = 0.003; Figure 3D). Interestingly, the patients’ mean BMI was not affected by smoking (Figure 3E). Despite the younger age, the average number of comorbidities was comparable between smokers (3.40 ± 0.15; 3.11–3.70) and non-smokers (3.59 ± 0.14; 3.33–3.86; Figure 3F).

### 3.4. Cytokine Levels are Altered in Smokers’ Blood

**Figure 4:**
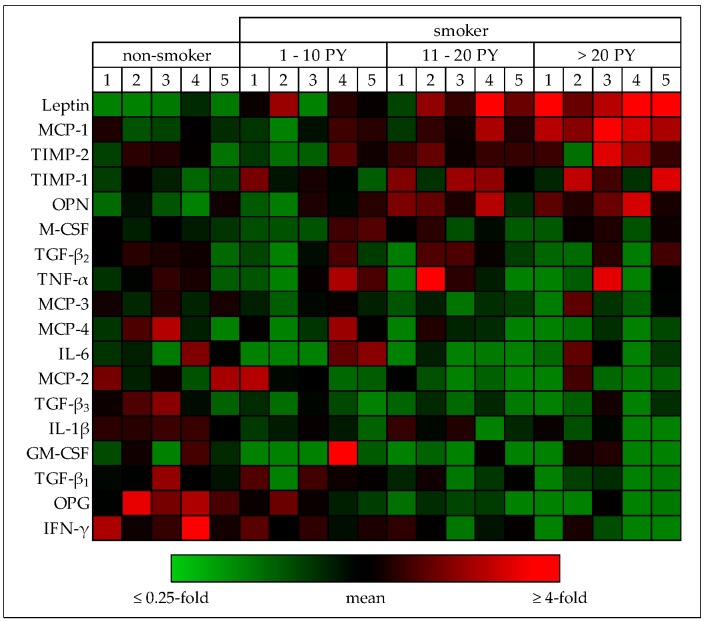
Effect of smoking on blood circulating factors. Relative cytokine levels in (pre-surgical) serum samples from non-smokers (0 PY), light smokers (1–10 PY), moderate smokers (11–20 PY) and heavy smokers (>20 PY) were determined with the help of the RayBio® Human Cytokine Array C5 (*N* = 5 per group). For the heat map, signal intensities were first normalized to the internal (positive) control followed by a second normalization to the mean signal intensity of each cytokine. Under-represented cytokines are colored green; over-represented cytokines are colored red.

In order get an idea on the possible regulatory mechanisms involved, we screened cytokine levels in the blood (pre-surgical) of patients receiving total joint arthroplasties (five each of non-smokers, light smokers: 1–10 PY, moderate smokers: 11–20 PY, and heavy smokers: >20 PY). Relative cytokine levels were determined with the help of the RayBio® Human Cytokine Array C5. As expected, blood serum levels of the appetite suppressant leptin and the pro-oxidative MCP-1 were increased with increasing number of pack-years. Similarly, blood serum levels of regulators of tissue integrity e.g. OPN (osteopontin favors adherence of osteoclasts) and the tissue inhibitors of metalloproteinases TIMP-1 and -2, were elevated in smokers. On the contrary, cytokine levels of pro-inflammatory IL-6, IL-1β, and IFN-γ were decreased in smokers. Blood serum levels of the immune regulatory TGF-β1 also decreased with increasing number of pack-years. Osteoprotegerin (OPG), the soluble decoy receptor of receptor activator of nuclear factor kappa-B ligand (sRANKL), was also decreased in smokers. These effects were more pronounced the more the patients smoked (Figure 4), suggesting that smokers have an imbalance in osteoblast-osteoclast function and a suppressed immune response.

### 3.5. Decreased Osteoblast Activity in Smokers Following Surgery

**Figure 5:**
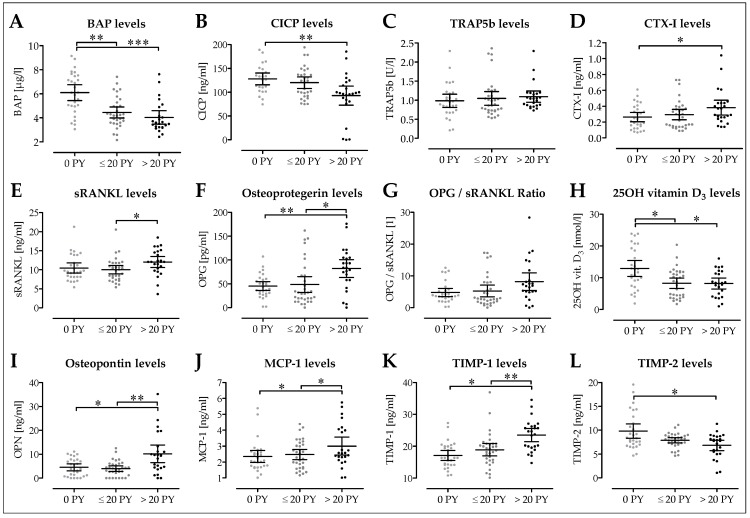
Effect of smoking on serum levels of markers for bone formation and resorption. The analysis differentiated between non-smokers (0 PY; *N* = 14), moderate smokers (1–20 PY; *N* = 16) and heavy smokers (>20 PY; *N* = 12). All ELISAs were performed in duplicates. As bone formation markers, serum levels of (**A**) bone specific alkaline phosphatase (BAP) and (**B**) type I C-terminal collagen pro-peptide (CICP) were determined. As bone resorption markers, serum levels of (**C**) tartrate-resistant acidic phosphatase (TRAP5b) and (**D**) C-terminal telo-peptide of type I collagen (CTX-I) were determined. As regulators for osteoclastogenesis, serum levels of (**E**) soluble receptor activator of nuclear factor kappa-B ligand (sRANKL), (**F**) Osteoprotegerin (OPG), (**G**) the resulting OPG:sRANKL ratio, and (**H**) the regulatory 25OH vitamin D3 were determined. (**I**) Osteopontin serum levels were determined as a marker for osteoclast adherence. (**J**) Monocyte chemoattractant protein-1 (MCP-1/CCL2) serum levels were determined as a stress marker. In addition, serum levels of the tissue inhibitor of metalloproteinases (**K**) TIMP-1 and (**L**) TIMP-2 were determined as an indicator for tissue turnover. * *p* < 0.05, ** *p* < 0.01, and *** *p* < 0.001 as indicated.

We first quantified blood serum levels of osteoblasts and osteoclast markers in our patients two days following surgery. Blood serum levels of bone-specific alkaline phosphatase (BAP) were significantly higher in non-smokers (6.10 ± 0.32 µg/L; 5.44–6.76 µg/L) than smokers, with heavy smokers showing lower BAP levels (4.04 ± 0.27 µg/L; 3.47–4.59 µg/L; *p* < 0.001) than moderate smokers (4.45 ± 0.22 µg/L; 3.99–4.90 µg/L; *p* = 0.003; Figure 5A). In line with this, blood serum levels of the bone formation marker type I C-terminal collagen pro-peptide (CICP) decreased with increasing number of pack-years (127.9 ± 6.0 ng/mL; 115.4–140.3 ng/mL vs. 120.0 ± 5.9 ng/mL; 107.8–132.1 ng/mL vs. 92.7 ± 9.7 ng/mL; 72.7–112.8 ng/mL; *p* = 0.004; Figure 5B). Interestingly, blood serum levels of the osteoclast marker tartrate-resistant acidic phosphatase (TRAP5b) and the bone resorption marker C-terminal telo-peptide of type I collagen (CTX-I) were not significantly affected by smoking (Figure 5C,D). Blood serum levels of both sRANKL and its decoy receptor OPG increased with increasing number of pack-years, such that the resulting OPG:sRANKL ratio was not affected by smoking (Figure 5E–G). This is interesting, as its regulator, 25(OH) vitamin D_3_, was significantly decreased in blood of smokers (8.3 ± 0.8 nmol/L; 6.6–9.9 nmol/L; *p* = 0.013, and 8.2 ± 0.8 nmol/L; 6.5–9.9 nmol/L, *p* = 0.012) when compared to the blood of non-smokers (12.9 ± 1.2 nmol/L; 10.4–15.4; Figure 5H). It is noteworthy that blood serum levels of OPN, which favors osteoclast adherence, were significantly increased in heavy smokers (10.2 ± 1.8 ng/ml; 6.5–13.8 ng/mL) when compared to non-smokers (4.6 ± 0.7 ng/ml; 3.1–6.0 ng/mL; *p* = 0.016) or moderate smokers (4.0 ± 0.6 ng/mL; 2.8–5.1 ng/mL; *p* = 0.002, Figure 5I). As expected, blood serum levels of MCP-1, a marker increased by oxidative stress, is significantly increased in heavy smokers (3.0 ± 0.3 ng/mL; 2.4–3.6 ng/mL) when compared to non-smokers (2.4 ± 0.2 ng/mL; 2.0–2.7 ng/mL; *p* = 0.045) or moderate smokers (2.5 ± 0.2 ng/mL; 2.2–2.8 ng/mL; *p* = 0.026, Figure 5J). In line with the cytokine array, blood serum levels of TIMP-1 were significantly increased in heavy smokers (23.5 ± 1.0 ng/mL; 21.4–25.6 ng/mL) when compared to non-smokers (17.1 ± 0.8 ng/mL; 15.6–18.7 ng/mL; *p* < 0.001) or moderate smokers (18.9 ± 0.9 ng/mL; 17.0–20.8 ng/mL; *p* = 0.002; Figure 5K). In contrast, blood serum levels of TIMP-2 were significantly decreased in heavy smokers (6.8 ± 0.5 ng/mL; 5.7–7.9 ng/mL) when compared to non-smokers (9.8 ± 0.7 ng/ml; 8.3–11.3 ng/mL; *p* = 0.020) or moderate smokers (7.9 ± 0.3 ng/mL; 7.3–8.4 ng/mL; Figure 5L).

### 3.6. Pro-Inflammatory Cytokine Levels are Decreased in Smokers Following Surgery

**Figure 6:**
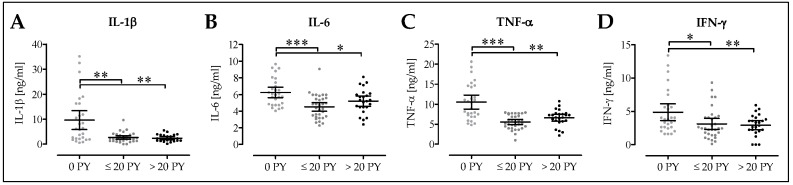
Effect of smoking on serum levels of inflammatory markers. The analysis differentiated between non-smokers (0 PY; *N* = 14), moderate smokers (1–20 PY; *N* = 16) and heavy smokers (>20 PY; *N* = 12). All ELISAs were performed in duplicates. Serum levels of (**A**) interleukin 1 beta (IL-1β), (**B**) interleukin 6 (IL-6), (**C**) tumor necrosis factor alpha (TNF-α), and (**D**) interferon gamma (IFN-γ) were determined. * *p* < 0.05, ** *p* < 0.01, and *** *p* < 0.001 as indicated.

In the next step, we quantified blood serum levels of pro-inflammatory cytokines in our patients two days following surgery. IL-1β serum levels were significantly lower in moderate smokers (2.6 ± 0.3 ng/mL; 1.9–3.3 ng/mL; *p* = 0.005) and heavy smokers (2.4 ± 0.3 ng/mL; 1.8–3.0 ng/mL; *p* = 0.002) when compared to non-smokers (9.7 ± 1.8 ng/mL; 5.9–13.4 ng/mL; Figure 6A). IL-6 blood serum levels were lower in moderate smokers (4.3 ± 0.3 ng/ml; 4.0–5.0 ng/mL; *p* < 0.001) and heavy smokers (5.2 ± 0.3 ng/mL; 4.6–5.8 ng/mL; *p* = 0.047) than in non-smokers (6.3 ± 0.3 ng/mL; 5.6–6.9 ng/mL; Figure 6B). Similarly, TNF-α blood serum levels were significantly lower in moderate smokers (5.5 ± 0.3 ng/mL; 4.9–6.2 ng/mL; *p* < 0.001) and heavy smokers (6.6 ± 0.4 ng/mL; 5.7–7.5 ng/mL; *p* = 0.002) than in non-smokers (10.5 ± 0.9 ng/mL; 8.8–12.3 ng/mL; Figure 6C). Blood serum levels of IFN-γ were significantly lower in heavy smokers (2.9 ± 0.3 ng/mL; 2.2–3.6 ng/mL; *p* = 0.008) and moderate smokers (3.1 ± 0.4 ng/ml; 2.3–3.9 ng/mL; *p* = 0.021) than in non-smokers (4.9 ± 0.6 ng/mL; 3.6–6.1 ng/mL; Figure 6D).

## 4. Discussion

Despite frequent reports on smoking as a risk factor for osteoporosis, fragility fractures, and associated post-surgical complication (e.g., delayed/impaired healing, infections, or revision surgeries) [3,4,5,6,7,8,9], little is known about the underlying mechanisms. Consequently, handling of smokers during and after musculoskeletal surgeries remains a huge challenge, as no specific treatment strategies exist for these patients. Thus, we set out to better characterize the possible underlying mechanisms that might cause delayed or impaired healing and infections in smokers receiving a TJA in order to propose possible supportive treatment strategies.

In our study cohort, which consisted of 817 patients that received a TJA (primary or revision), smoking was confirmed as a major risk factor for complications (infections, disturbed wound healing, required revision surgery, thrombosis, and/or death). Compared to patients at risk for malnutrition (NRS ≥ 3) and patients with daily alcohol intake, the frequency of complications was higher in smokers. Furthermore, there was a positive correlation between the risk for complications and the amount of smoked cigarettes, which is in line with epidemiological reports [30]. Looking closer at the smokers in our study cohort revealed a higher proportion of men than women in this subgroup. The obtained female to male ratio of approx. 0.43 is lower than that reported for Germany by the World Health Organization (approx. 0.6) [41]. However, this difference might be due to the overall higher amount of men (*N* = 445) than women (*N* = 343) in our study cohort. Although representative for Germany, the female to male ratio among orthopedic/trauma patients might be different in other countries, as the reported smoking behavior varies strongly [41]. Interestingly, in our patients, the mean number of comorbidities seemed to not be increased in smokers. However, this might be explained by the stringing difference in the patients’ age. Smokers in our study population were significantly younger than non-smokers (on average 5.4 years), suggesting that smokers are at higher risk for fragility fractures at a much younger age than non-smokers. This is in line with the five-year longitudinal study of Rudang et al. reporting impaired bone mass development and associated higher risk for fragility fractures in young adult men [3]. An extended meta-analysis by Pearson et al. showed not only significantly delayed fracture healing but also a higher frequency of non-unions after fracture, spinal fusion, osteotomy, or arthrodesis in smokers [7]. This finding is fostered by the retrospective study of Hess et al. reporting a higher frequency of post-surgical tenderness, wrist stiffness, non-unions, and revision surgeries in smokers with distal radius fractures (when compared to non-smokers) [6].

Thus, it is indisputable that smoking affects bone health, increases fracture risk, impairs bone healing, and increases the risk for complications. Despite many novel implants and great progresses in surgical techniques, there is still a high frequency of complications in smokers following trauma/orthopedic surgery. As smoking cannot be forbidden in these patients, there is a great need for specific treatment strategies in order to reduce associated complications, e.g., altered antibiosis, immune-modulators, and drugs favoring bone formation or inhibiting bone resorption. However, to establish such secondary treatment strategies, the underlying mechanisms have to be better understood.

Reports on periodontitis patients propose increased bone resorption in the jaw of smokers, as these patients frequently show decreased OPG:sRANKL ratios [11,12,13,14,15,16,17]. This is not supported by our finding. Although the cytokine array showed pre-surgically decreased OPG levels in our smokers, quantification of the OPG levels with ELISA two days post-surgery showed contrary results, suggesting the stimulation of OPG expression by the surgery in heavy smokers. Similar results hold for the associated sRANKL, which is strongly induced in heavy smokers two days following surgery, such that the OPG:sRANKL ratio was not significantly altered. These partly contradictory results might be due to the fact that the oral cavity is more affected by direct contact to the cigarette smoke than the skeleton where the effectors have to reach the bone via the blood stream. Considering the stable OPG:sRANKL ratios, it was not surprising that TRAP5b serum levels were comparable between smokers and non-smokers in our patients. Expression of sRANKL is known to be upregulated following orthopedic surgery as a surgery-induced oxidative stress response and a possible immune response towards the implanted material [42]. Just recently, Blaschke et al. showed that sRANKL expression was induced in mesenchymal cells by a combination of the inflammatory cytokines IL-1β, IL-6, and TNF-α [43]. Interestingly, serum levels of these pro-inflammatory markers were reduced in our smokers, which is in line with other reports stating a general immune suppression in smokers [35,36,37]. This might explain why smokers are more susceptible to infections [30,36]. 25(OH)D_3_ is a well described regulator of MCP-1, OPG, and sRANKL expression [26,27]. Thus, decreased 25(OH)D_3_ serum levels in our cohort of smokers smokers go well together with the increased MCP-1 serum levels in these patients. Although sRANKL expression is increased by 25(OH)D_3_ in vitro [27], 25(OH)D_3_ supplementation improves bone mineral density in patients via inhibition of osteoclasts [26,29]. Thus, it is not surprising that the bone resorption marker CTX-I was mildly induced in heavy smokers with decreased 25(OH)D_3_ serum levels. General supplementation of 25(OH)D_3_ in these patients has to be carefully considered, as 25(OH)D_3_ is also known as a potent regulator of immune responses [32] with strong immune-suppressive action [44]. Further studies are necessary to investigate whether supplementation of 25(OH)D_3_ in immune-suppressed smokers increases their risk for infections.

Serum levels of the oxidative stress marker MCP-1 were increased in our heavy smokers, which is in accordance with other reports [45,46]. Accumulation of Reactive Oxygen Species (ROS) can directly induce formation of osteoclasts from mononuclear cells [47,48] and also by upregulation of MCP-1, a strong inducer of osteoclast fusion and osteoclastogenesis [22,23,24]. MCP-1 can be induced not only by oxidative stress but also by sRANKL [21,24], which was also increased in these patients. Increased serum levels of OPN, a glycoprotein in bone tissue which functions as an anchor for osteoclasts [38], might further favor osteoclastogenesis in heavy smokers. Thus, it was not surprising that serum levels of CTX-I were mildly induced in heavy smokers, indicating increased bone resorption. Furthermore, we found a smoking-dependent imbalance in TIMP-1 and TIMP-2 serum levels, which may affect matrix resorption via matrix metalloproteinases (MMPs) [49]. Interestingly, TIMP-1 knock-out mice showed significantly stronger inflammatory responses after injury than wild-type mice, suggesting that TIMP-1 may restrict inflammation following injury [50]. Thus, it is feasible that increased TIMP-1 levels in smokers might contribute to the observed decrease in inflammatory response following surgery in these patients. TIMP-2, which was decreased in our smokers, is supposed to play a crucial role in protecting the extracellular matrix (ECM) from proteolysis during fracture healing [51]. Its overexpression in turn may cause pathophysiological ECM accumulation in patients with Dupuytren’s disease [52].

However, smoking effects were more pronounced on osteoblastic cells. BAP serum levels were already significantly reduced in moderate smokers, indicating reduced bone formation. This finding is underlined by the reduced serum levels of the bone formation marker CICP in these patients. Experiments with bone marrow-derived mesenchymal stem cells show the strong inhibitory effects of cigarette smoke extract on the cells osteogenic differentiation [18,19]. This effect was not mediated directly by nicotine or its more stable metabolite cotinine [18] but to a big part by oxidative stress caused by reactive substances in cigarette smoke formed in the burning process. When oxidative stress from cigarette smoke extract accumulates, it strongly affects the viability of primary human osteoblasts [20].

Thus, alternative smoking devices, e.g., e-cigarettes or tobacco heat systems, are advertised as potentially less harmful alternatives [53]. However, long term studies showing the effect of these potential alternatives to cigarettes on bone are still missing. Therefore, smokers are still encouraged to abstain from smoking by studies on patients with acute fracture surgery, which showed less complications, when offered a standardized smoking cessation program for six weeks following surgery [54]. As acute intervention has already shown promising results, further studies on pre-operative smoking cessation followed, which similarly showed reduced complication rates in patients with pre-operative smoking cessation [55,56]. Active intervention, e.g., with standardized smoking cessation programs, kept patients from smoking for longer times [57]. Offering of alternative products, e.g., e-cigarettes or tobacco heat systems, were helpful in reducing acute cravings for cigarettes [58]. However, the effects of this kind of intervention on the clinical outcome of orthopedic/trauma surgery have yet to be investigated.

## 5. Conclusions

Our data confirm that smoking is a major risk factor for complications following TJA, even at an early age. This holds true for primary TJA, where the overall rate of smokers is lower, as well as for revision TJA, where the overall rate of smokers was significantly higher. Thus, smokers should be encouraged to abstain from smoking to improve the outcome of orthopedic surgeries, especially while no specific treatment strategies are available for these patients.

We could identify alterations in serum levels suggesting a mild increase in bone resorption in heavy smokers. Bone formation was already strongly affected in mild smokers. Thus, bone anabolic drugs might be feasible to stimulate bone formation in smokers following orthopedic or trauma surgery. Post-surgical activation of the immune system is strongly reduced in smokers, suggesting an impaired immune response in these patients, which makes them potentially susceptible for infections. Therefore, general administration of 25(OH)D_3_, which is a strong immune suppressant, to stabilize bone metabolism should be carefully deliberated. We further identified an imbalance in TIMP-1 and TIMP-2 serum levels in smokers, which might represent novel regulators and thus therapeutic targets for both bone regeneration and immune responses in these patients.

## Figures and Tables

**Table 1:** Overview on the performed enzyme linked immunosorbent assays (ELISAs).

Target	Function	ELISA Kit		Dilution Factor
		Order No.	Company	
25(OH)D_3_	25OH vitamin D_3_	AC-57DF1	IDS	-
BAP	Osteoblast activity	AC-20F1	IDS	-
TRAP5b	Osteoclast activity	SB-TR201A	IDS	-
CTX-I	Bone resorption	AC-57SF1	IDS	3
CICP	Collagen synthesis	8003	TecoMedical	12.5
MCP-1	Stress marker	900-K31	Peprotech	20
sRANKL	Favors osteoclastogenesis	900-K142	Peprotech	20
TIMP-1	Inhibitor for MMPs	900-M438	Peprotech	20
IL-1β	Inflammatory marker	900-K95	Peprotech	10
IL-6	Inflammatory marker	900-K16	Peprotech	10
TNF-α	Inflammatory marker	900-K25	Peprotech	10
IFN-γ	Inflammatory marker	900-K27	Peprotech	10
TIMP-2	Inhibitor for MMPs	ELH-TIMP2	BioCat	100
OPG	Inhibitor for sRANKL	ELH-OPG	BioCat	20
OPN	Anchor for osteoclasts	ELH-OPN	BioCat	20

25(OH)D_3_: 25 hydroxy vitamin D_3_; BAP: bone specific alkaline phosphatase; TRAP5b: tartrate-resistant acidic phosphatase; CTX-I: C-terminal telo-peptide of type I collagen; CICP: type I C-terminal collagen pro-peptide; MCP-1: Monocyte chemoattractant protein-1; sRANKL: soluble receptor activator of nuclear factor kappa-B ligand; TIMP-1 and TIMP-2: tissue inhibitor of metalloproteinases; OPG: Osteoprotegerin; OPN: Osteopontin; IL-1β: interleukin 1 beta; IL-6: interleukin 6; TNF-α: tumor necrosis factor alpha; IFN-γ: interferon gamma. Order No.: Order number of the company.

**Table 2:** Overview on the study population.

			w/o Complications	w/ Complications	All Patients	*p*-Value
**Primary Athroplasties**	Age (a)		63.1 ± 14.9 (61.7–64.5)	60.8 ± 14.3 (57.5–64.0)	62.7 ± 14.8 (61.5–64.0)	0.106
	Gender distribution	Male	49.2% (*N* = 212)	65.8% (*N* = 52)	65.4% (*N* = 264)	0.022
		Female	50.8% (*N* = 219)	34.2% (*N* = 27)	34.6% (*N* = 246)	
		BMI (kg/m^2^)	28.1 ± 5.1 (27.7–28.6)	29.0 ± 5.8 (27.7–30.3)	28.3 ± 5.2 (27.8–28.7)	0.263
		Number of comorbidities	3.47 ± 2.65 (3.22–3.73)	3.86 ± 3.49 (3.08–4.64)	3.54 ± 2.80 (3.29–3.78)	0.821
		Number of drugs	3.50 ± 3.25 (3.19–3.81)	4.19 ± 4.66 (3.15–5.23)	3.61 ± 3.51 (3.30–3.92)	0.844
		Frequency of malaise (%)	9.3% (*N* = 40)	17.7% (*N* = 14)	10.6% (*N* = 54)	0.097
**Revision Athroplasties**	Age (a)		60.3 ± 16.7 (58.0–62.7)	59.1 ± 15.2 (55.8–62.5)	60.0 ± 16.3 (58.1–61.9)	0.437
	Gender distribution	Male	65.2% (*N* = 129)	65.0% (*N* = 52)	65.1% (*N* = 181)	1.000
		Female	34.8% (*N* = 69)	35.0% (*N* = 28)	34.9% (*N* = 97)	
		BMI (kg/m^2^)	28.2 ± 5.8 (27.4–29.1)	29.0 ± 6.8 (27.5–30.5)	28.5 ± 6.1 (27.7–29.2)	0.809
		Number of comorbidities	3.34 ± 2.87 (3.94–3.74)	3.63 ± 3.12 (2.93–4.32)	3.42 ± 2.94 (3.07–3.77)	0.574
		Number of drugs	3.55 ± 3.39 (3.08–4.03)	3.56 ± 3.62 (2.75–4.37)	3.56 ± 3.45 (3.14–3.97)	0.795
		Frequency of malaise (%)	8.6% (*N* = 17)	31.3% (*N* = 25)	15.1% (*N* = 42)	<0.001

## References

[1] Daniell H.W. (1976). Osteoporosis of the slender smoker. Vertebral compression fractures and loss of metacarpal cortex in relation to postmenopausal cigarette smoking and lack of obesity. Arch. Intern. Med..

[2] Benson B.W., Shulman J.D. (2005). Inclusion of tobacco exposure as a predictive factor for decreased bone mineral content. Nicotine Tob. Res..

[3] Rudang R., Darelid A., Nilsson M., Nilsson S., Mellstrom D., Ohlsson C., Lorentzon M. (2012). Smoking is associated with impaired bone mass development in young adult men: A 5-year longitudinal study. J. Bone Miner. Res..

[4] Sloan A., Hussain I., Maqsood M., Eremin O., El-Sheemy M. (2010). The effects of smoking on fracture healing. Surgeon.

[5] Scolaro J.A., Schenker M.L., Yannascoli S., Baldwin K., Mehta S., Ahn J. (2014). Cigarette smoking increases complications following fracture: A systematic review. J. Bone Joint Surg. Am..

[6] Hess D.E., Carstensen S.E., Moore S., Dacus A.R. (2018). Smoking increases postoperative complications after distal radius fracture fixation: A review of 417 patients from a level 1 trauma center. Hand (N Y).

[7] Pearson R.G., Clement R.G., Edwards K.L., Scammell B.E. (2016). Do smokers have greater risk of delayed and non-union after fracture, osteotomy and arthrodesis? A systematic review with meta-analysis. BMJ Open.

[8] Abate M., Vanni D., Pantalone A., Salini V. (2013). Cigarette smoking and musculoskeletal disorders. Muscles Ligaments Tendons J..

[9] Kanis J.A., Johnell O., Oden A., Johansson H., De Laet C., Eisman J.A., Fujiwara S., Kroger H., McCloskey E.V., Mellstrom D. (2005). Smoking and fracture risk: A meta-analysis. Osteoporos. Int..

[10] Qiu F., Liang C.L., Liu H., Zeng Y.Q., Hou S., Huang S., Lai X., Dai Z. (2017). Impacts of cigarette smoking on immune responsiveness: Up and down or upside down?. Oncotarget.

[11] Behfarnia P., Saied-Moallemi Z., Javanmard S.H., Naseri R. (2016). Serum, saliva, and gcf concentration of rankl and osteoprotegerin in smokers versus nonsmokers with chronic periodontitis. Adv. Biomed. Res..

[12] Bostrom E.A., Kindstedt E., Sulniute R., Palmqvist P., Majster M., Holm C.K., Zwicker S., Clark R., Onell S., Johansson I. (2015). Increased eotaxin and mcp-1 levels in serum from individuals with periodontitis and in human gingival fibroblasts exposed to pro-inflammatory cytokines. PLoS One.

[13] Belibasakis G.N., Bostanci N. (2012). The rankl-opg system in clinical periodontology. J. Clin. Periodontol..

[14] Ozcaka O., Nalbantsoy A., Kose T., Buduneli N. (2010). Plasma osteoprotegerin levels are decreased in smoker chronic periodontitis patients. Aust. Dent. J..

[15] Buduneli N., Buduneli E., Kutukculer N. (2009). Interleukin-17, rankl, and osteoprotegerin levels in gingival crevicular fluid from smoking and non-smoking patients with chronic periodontitis during initial periodontal treatment. J. Periodontol..

[16] Buduneli N., Biyikoglu B., Sherrabeh S., Lappin D.F. (2008). Saliva concentrations of rankl and osteoprotegerin in smoker versus non-smoker chronic periodontitis patients. J. Clin. Periodontol..

[17] Lappin D.F., Sherrabeh S., Jenkins W.M., Macpherson L.M. (2007). Effect of smoking on serum rankl and opg in sex, age and clinically matched supportive-therapy periodontitis patients. J. Clin. Periodontol..

[18] Aspera-Werz R.H., Ehnert S., Heid D., Zhu S., Chen T., Braun B., Sreekumar V., Arnscheidt C., Nussler A.K. (2018). Nicotine and cotinine inhibit catalase and glutathione reductase activity contributing to the impaired osteogenesis of scp-1 cells exposed to cigarette smoke. Oxid. Med. Cell Longev..

[19] Sreekumar V., Aspera-Werz R., Ehnert S., Strobel J., Tendulkar G., Heid D., Schreiner A., Arnscheidt C., Nussler A.K. (2018). Resveratrol protects primary cilia integrity of human mesenchymal stem cells from cigarette smoke to improve osteogenic differentiation in vitro. Arch. Toxicol..

[20] Ehnert S., Braun K.F., Buchholz A., Freude T., Egana J.T., Schenck T.L., Schyschka L., Neumaier M., Dobele S., Stockle U. (2012). Diallyl-disulphide is the effective ingredient of garlic oil that protects primary human osteoblasts from damage due to cigarette smoke. Food Chem..

[21] Deshmane S.L., Kremlev S., Amini S., Sawaya B.E. (2009). Monocyte chemoattractant protein-1 (mcp-1): An overview. J. Interferon Cytokine Res..

[22] Morrison N.A., Day C.J., Nicholson G.C. (2014). Dominant negative mcp-1 blocks human osteoclast differentiation. J. Cell Biochem..

[23] Miyamoto K., Ninomiya K., Sonoda K.H., Miyauchi Y., Hoshi H., Iwasaki R., Miyamoto H., Yoshida S., Sato Y., Morioka H. (2009). Mcp-1 expressed by osteoclasts stimulates osteoclastogenesis in an autocrine/paracrine manner. Biochem. Biophys. Res. Commun..

[24] Kim M.S., Day C.J., Morrison N.A. (2005). Mcp-1 is induced by receptor activator of nuclear factor-{kappa}b ligand, promotes human osteoclast fusion, and rescues granulocyte macrophage colony-stimulating factor suppression of osteoclast formation. J. Biol. Chem..

[25] Khan U.A., Hashimi S.M., Bakr M.M., Forwood M.R., Morrison N.A. (2016). Ccl2 and ccr2 are essential for the formation of osteoclasts and foreign body giant cells. J. Cell. Biochem..

[26] Wintermeyer E., Ihle C., Ehnert S., Stockle U., Ochs G., de Zwart P., Flesch I., Bahrs C., Nussler A.K. (2016). Crucial role of vitamin d in the musculoskeletal system. Nutrients.

[27] Takahashi N., Udagawa N., Suda T. (2014). Vitamin d endocrine system and osteoclasts. Bonekey Rep..

[28] Wang Y.C., Hsieh C.C., Kuo H.F., Tsai M.K., Yang S.N., Kuo C.H., Lee M.S., Hung C.H. (2014). Effect of vitamin d3 on monocyte chemoattractant protein 1 production in monocytes and macrophages. Acta Cardiol. Sin..

[29] Baldock P.A., Thomas G.P., Hodge J.M., Baker S.U., Dressel U., O’Loughlin P.D., Nicholson G.C., Briffa K.H., Eisman J.A., Gardiner E.M. (2006). Vitamin d action and regulation of bone remodeling: Suppression of osteoclastogenesis by the mature osteoblast. J. Bone Miner. Res..

[30] Knapik J.J., Bedno S.A. (2018). Epidemiological evidence and possible mechanisms for the association between cigarette smoking and injuries (part 1). J. Spec. Oper. Med..

[31] Bon J., Zhang Y., Leader J.K., Fuhrman C., Perera S., Chandra D., Bertolet M., Diergaarde B., Greenspan S.L., Sciurba F.C. (2018). Radiographic emphysema, circulating bone biomarkers, and progressive bone mineral density loss in smokers. Ann. Am. Thorac. Soc..

[32] Sassi F., Tamone C., D’Amelio P. (2018). Vitamin d: Nutrient, hormone, and immunomodulator. Nutrients.

[33] Javed F., Al-Kheraif A.A., Al Amri M.D., Alshehri M., Vohra F., Al-Askar M., Malmstrom H., Romanos G.E. (2015). Periodontal status and whole salivary cytokine profile among smokers and never-smokers with and without prediabetes. J. Periodontol..

[34] Suzuki N., Nakanishi K., Yoneda M., Hirofuji T., Hanioka T. (2016). Relationship between salivary stress biomarker levels and cigarette smoking in healthy young adults: An exploratory analysis. Tob. Induc. Dis..

[35] Lugade A.A., Bogner P.N., Thatcher T.H., Sime P.J., Phipps R.P., Thanavala Y. (2014). Cigarette smoke exposure exacerbates lung inflammation and compromises immunity to bacterial infection. J. Immunol..

[36] Goncalves R.B., Coletta R.D., Silverio K.G., Benevides L., Casati M.Z., da Silva J.S., Nociti F.H. (2011). Impact of smoking on inflammation: Overview of molecular mechanisms. Inflamm. Res..

[37] Chen H., Cowan M.J., Hasday J.D., Vogel S.N., Medvedev A.E. (2007). Tobacco smoking inhibits expression of proinflammatory cytokines and activation of il-1r-associated kinase, p38, and nf-kappab in alveolar macrophages stimulated with tlr2 and tlr4 agonists. J. Immunol..

[38] Schell H., Duda G.N., Peters A., Tsitsilonis S., Johnson K.A., Schmidt-Bleek K. (2017). The haematoma and its role in bone healing. J. Exp. Orthop..

[39] Ihle C., Freude T., Bahrs C., Zehendner E., Braunsberger J., Biesalski H.K., Lambert C., Stockle U., Wintermeyer E., Grunwald J. (2017). Malnutrition - an underestimated factor in the inpatient treatment of traumatology and orthopedic patients: A prospective evaluation of 1055 patients. Injury.

[40] Ihle C., Bahrs C., Freude T., Bickel M., Spielhaupter I., Wintermeyer E., Stollhof L., Grunwald L., Ziegler P., Pscherer S. (2017). Malnutrition in elderly trauma patients - comparison of two assessment tools. Z. Orthop. Unfall..

[41] Hitchman S.C., Fong G.T. (2011). Gender empowerment and female-to-male smoking prevalence ratios. Bull. World Health Organ..

[42] Kapasa E.R., Giannoudis P.V., Jia X., Hatton P.V., Yang X.B. (2017). The effect of rankl/opg balance on reducing implant complications. J. Funct. Biomater..

[43] Blaschke M., Koepp R., Cortis J., Komrakova M., Schieker M., Hempel U., Siggelkow H. (2018). Il-6, il-1beta, and tnf-alpha only in combination influence the osteoporotic phenotype in crohn’s patients via bone formation and bone resorption. Adv. Clin. Exp. Med..

[44] Calton E.K., Keane K.N., Newsholme P., Soares M.J. (2015). The impact of vitamin d levels on inflammatory status: A systematic review of immune cell studies. PLoS One.

[45] Komiyama M., Takanabe R., Ono K., Shimada S., Wada H., Yamakage H., Satoh-Asahara N., Morimoto T., Shimatsu A., Takahashi Y. (2018). Association between monocyte chemoattractant protein-1 and blood pressure in smokers. J. Int. Med. Res..

[46] Di Stefano A., Coccini T., Roda E., Signorini C., Balbi B., Brunetti G., Ceriana P. (2018). Blood mcp-1 levels are increased in chronic obstructive pulmonary disease patients with prevalent emphysema. Int. J. Chron. Obstruct. Pulmon. Dis..

[47] Srinivasan S., Koenigstein A., Joseph J., Sun L., Kalyanaraman B., Zaidi M., Avadhani N.G. (2010). Role of mitochondrial reactive oxygen species in osteoclast differentiation. Ann. N. Y. Acad. Sci..

[48] Lee N.K., Choi Y.G., Baik J.Y., Han S.Y., Jeong D.W., Bae Y.S., Kim N., Lee S.Y. (2005). A crucial role for reactive oxygen species in rankl-induced osteoclast differentiation. Blood.

[49] Brew K., Dinakarpandian D., Nagase H. (2000). Tissue inhibitors of metalloproteinases: Evolution, structure and function. Biochim. Biophys. Acta.

[50] Kim K.H., Burkhart K., Chen P., Frevert C.W., Randolph-Habecker J., Hackman R.C., Soloway P.D., Madtes D.K. (2005). Tissue inhibitor of metalloproteinase-1 deficiency amplifies acute lung injury in bleomycin-exposed mice. Am. J. Respir. Cell Mol. Biol..

[51] Lieu S., Hansen E., Dedini R., Behonick D., Werb Z., Miclau T., Marcucio R., Colnot C. (2011). Impaired remodeling phase of fracture repair in the absence of matrix metalloproteinase-2. Dis. Model. Mech..

[52] Arpino V., Brock M., Gill S.E. (2015). The role of timps in regulation of extracellular matrix proteolysis. Matrix Biol..

[53] Glantz S.A. (2018). Heated tobacco products: The example of iqos. Tob. Control..

[54] Nasell H., Adami J., Samnegard E., Tonnesen H., Ponzer S. (2010). Effect of smoking cessation intervention on results of acute fracture surgery: A randomized controlled trial. J. Bone Joint Surg. Am..

[55] Lindstrom D., Sadr Azodi O., Wladis A., Tonnesen H., Linder S., Nasell H., Ponzer S., Adami J. (2008). Effects of a perioperative smoking cessation intervention on postoperative complications: A randomized trial. Ann. Surg..

[56] Ring J., Shoaib A., Shariff R. (2017). Smoking cessation advice in limb reconstruction: An opportunity not to be missed. Injury.

[57] Villebro N.M., Pedersen T., Moller A.M., Tonnesen H. (2008). Long-term effects of a preoperative smoking cessation programme. Clin. Respir. J..

[58] Adriaens K., Gucht D.V., Baeyens F. (2018). Iqos(tm) vs. E-cigarette vs. Tobacco cigarette: A direct comparison of short-term effects after overnight-abstinence. Int. J. Environ. Res. Public Health.

